# DeepTumor: Framework for Brain MR Image Classification, Segmentation and Tumor Detection

**DOI:** 10.3390/diagnostics12112888

**Published:** 2022-11-21

**Authors:** Ghazanfar Latif

**Affiliations:** 1 Computer Science Department, Prince Mohammad Bin Fahd University, Khobar 34754, Saudi Arabia; glatif@pmu.edu.sa; 2 Department of Computer Sciences and Mathematics, Université du Québec à Chicoutimi, 555 boulevard de l’Université, Chicoutimi, QC G7H 2B1, Canada

**Keywords:** glioma tumor classification, tumor segmentation, neighboring FCM, deep learning, convolutional neural networks, tumor detection framework

## Abstract

The proper segmentation of the brain tumor from the image is important for both patients and medical personnel due to the sensitivity of the human brain. Operation intervention would require doctors to be extremely cautious and precise to target the brain’s required portion. Furthermore, the segmentation process is also important for multi-class tumor classification. This work primarily concentrated on making a contribution in three main areas of brain MR Image processing for classification and segmentation which are: Brain MR image classification, tumor region segmentation and tumor classification. A framework named DeepTumor is presented for the multistage-multiclass Glioma Tumor classification into four classes; Edema, Necrosis, Enhancing and Non-enhancing. For the brain MR image binary classification (Tumorous and Non-tumorous), two deep Convolutional Neural Network) CNN models were proposed for brain MR image classification; 9-layer model with a total of 217,954 trainable parameters and an improved 10-layer model with a total of 80,243 trainable parameters. In the second stage, an enhanced Fuzzy C-means (FCM) based technique is proposed for the tumor segmentation in brain MR images. In the final stage, an enhanced CNN model 3 with 11 hidden layers and a total of 241,624 trainable parameters was proposed for the classification of the segmented tumor region into four Glioma Tumor classes. The experiments are performed using the BraTS MRI dataset. The experimental results of the proposed CNN models for binary classification and multiclass tumor classification are compared with the existing CNN models such as LeNet, AlexNet and GoogleNet as well as with the latest literature.

## 1. Introduction

The study of automated diagnosis of brain tumors is an important subject for affected patients, doctors, technicians, and hospitals. For patients, early diagnosis can offer a better survival rate through early treatment and intervention. For doctors and technicians, it can offer a more accurate and faster way of diagnosis and treatment options. For hospitals and the general healthcare system, it reduces the cost of healthcare through an early diagnosis which means early intervention with less expensive treatment options. Automatic brain tumor detection plays an important role in assisting radiologists in diagnosing the brain tumor. Image segmentation plays an equally important role in identifying the location of the tumor [1,2]. Different classification and segmentation methods were presented in recent research studies for the detection of brain tumors. Through extensive analysis of previous research, it was found that most studies either suffer from or do not account for the most common problems of overfitting and lack of sufficiently sized datasets [3,4]. The overfitting problem occurs for many reasons, including a large number of hidden layers leading to extracting noise features that negatively affect the classifier performance. Various techniques have been proposed to tackle the overfitting problem: early stopping, training with more data, regularization, cross-validation, and dropout. Identifying the optimized deep learning model for brain MR images and glioma tumor classification is the main aim of this study. Here, it will be worth to mention that there are many types of primary brain tumors that exist and glioma tumor is most common type of brain tumor which is produced by the glial cells. The contribution of this research is an enhanced classification and segmentation methods which consist of a multistage process to classify the Glioma tumor into its multiclass; Necrosis, Edema, Enhancing and Non-Enhancing.

The work presented in this research is of interest to researchers in the field and medical personnel specialized in cancer treatment. This work primarily contributed to three main areas of brain MR Image processing for classification and segmentation. In the first stage, proposed significant contributions in the classification of MR images into Tumorous and Non-tumorous was presented. In the second stage, proposed techniques for the segmentation of the tumorous image were explained. In the third stage, proposed significant contributions for the classification of tumorous images into the four Glioma classes; Necrosis, Edema, Enhancing, and Non-enhancing were detailed [5].

The goal of this research is to propose an enhanced classification and segmentation techniques using deep learning models by tuning the deep learning parameters to avoid the overfitting problem and increase the classification and segmentation accuracy of binary and multiclass brain tumors. Recent developments in the computing field with high-speed multi-core processors and GPUs made it possible to explore image processing techniques that were put on the shelf previously because of their high-speed processing demand.

The rest of article is organized as follows; Section 2 discusses about the materials and methods. In Section 3, the experimental results and analysis is presented. In Section 4, the conclusion and future directions are provided.

### 1.1. Medical Image Modalities

**Figure 1:**
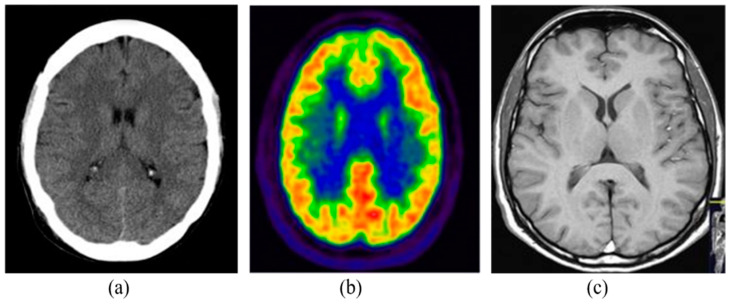
Brain imaging using different modalities: (**a**) CT, (**b**) PET, (**c**) MRI.

The medical imaging modalities define several methods to capture the image for the structure of some specific organs of the human body. Medical imaging has become an essential part of the clinical procedures due to its effective visualization and quantitative assessment. There are many different modalities of medical imaging available such as Computed Tomography (CT), Positron Emission Tomography (PET) and Magnetic Resonance Imaging (MRI) [6]. Some of the commonly and widely used imaging modalities are detailed in the following subsections. Figure 1 shows the samples of brain images captured from different imaging modalities.

### 1.2. Brain MRI

In brain MRI, magnetic fields produced by large magnets along with radio waves and a computing device are used to generate detailed information of the internal structure of the brain. There are three types of images produced by the brain MRI procedure and these types are based on the magnetic field strength and frequency of the waves. Changing the pulse order and image constraints, the following types of images are acquired: proton density (PD) weighted, longitudinal relaxation time (T1) weighted and transverse relaxation time (T2) weighted [7]. T1 images show the dark internal tissues of the brain whereas the T2 images indicate the bright tissues inside. PD images show the water and macromolecules inside the image [8].

A magnetic field is mainly used by brain MRI unlike radiations in CT scan or other techniques and can detect tissue swelling, infection and tumors inside the brain. The images obtained from MRI can be used for the analysis of the different types of brain abnormalities.

**Figure 2:**
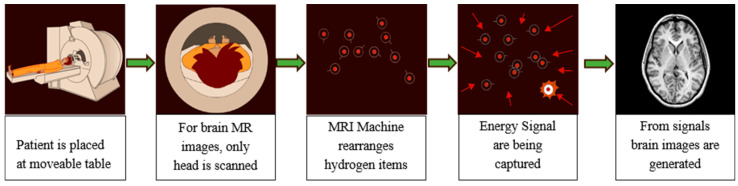
MR image capturing process from MRI Scanner.

In the brain MRI procedure, large magnets produce a magnetic field in the range 0.2 T to 7 T (average 1.5 T). The subject is placed inside this magnetic field and excited hydrogen atoms inside the body, due to the presence of water molecules, emit radio frequencies that are captured in the large enclosed area of the MRI scanner. These frequencies are used to generate the images inside a computing system. Various brain MR image modalities are obtained by changing the magnetic field strength. The large magnets’ coils are switched to on and off states to detect the timing information of the hydrogen atoms’ realignment into an equilibrium state. The process typically takes from 20 to 45 min to complete and is shown in Figure 2.

### 1.3. Brain MRI

Brain MRI provides the option to obtain different image modalities by changing the strength of the magnetic field and timing. Echo time in MR imaging is the time for which the radio frequencies emitted by the excited hydrogen atoms are measured. Repetition time is the time delay between two consecutive echo times. Changing the echo time and repetition time can have four different image modalities.

- T1: This modality has a small echo and repetition time. T1 provides a nice image contrast for the various healthy tissues inside the brain, i.e., gray matter, cerebrospinal fluid and white matter, etc.
- T2: It has a long time of echo and repetition time but slow image acquisition. It provides good contrast for the tumor surrounding tissues (edema).
- T1c: It is the same as T1, but a contrast agent is applied to enhance the contrast.
- FLAIR: It is used to nullify the signal from the fluid, suppress the effect of Cerebrospinal Fluid (CSF), and bring out the periventricular hyperintense lesion.

**Figure 3:**
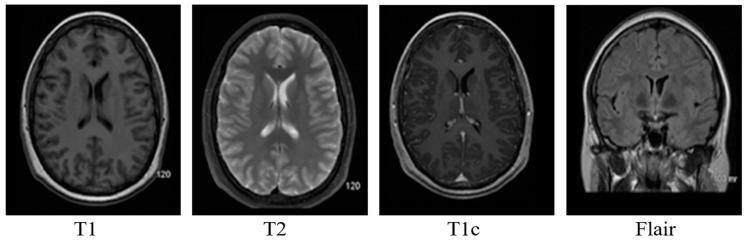
Pictorial view of brain MR Image modalities.

**Figure 4:**
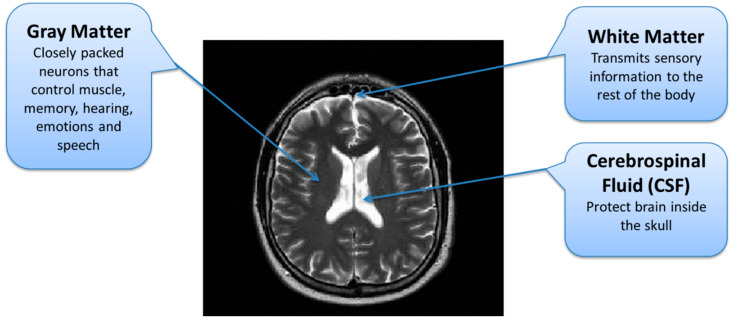
Brain MRI Tissues Types.

Brain MRI tumors have complicated structures and shapes, which makes the tumor classification and segmentation process more difficult using uni-modality. MRI machines provide an option to capture multimodality images with a more detailed representation of brain tissues [9]. During the MRI scan of a patient, the MRI machine produces different types of MRI sequences including T1, T2, T1c, and Flair, which are based on the Time to Echo (TE), Repetition Time (TR), brightness and contrast values. Figure 3 describes the four different brain MRI modalities and Figure 4 describes the three different types of healthy tissues inside the brain.

### 1.4. Convolutional Neural Network (CNN) for MR Image Analysis

The use of CNN for brain MR image classification is proposed with the use of small kernels for deep architectures. They achieved an average accuracy of 97.5%. The Deep CNN was applied to the BraTS 2015 dataset containing tumorous and non-tumorous images. Using the Deep CNN lowers the complexity and the computation time [10]. This study’s limitation is that it only classifies the images into tumorous and non-tumorous images and does not study the multimodal analysis of brain tumors. A 3D deep CNN architecture for brain MR image classification into LGG and HGG glioma brain tumor using the complete volumetric T1-Gado MRI sequence is proposed by Mzoughi et al. [11]. The proposed method merges both local and global features by utilizing deep networks with the use of small kernels. Preprocessing was done using adaptive contrast enhancement along with intensity normalization to over the data heterogeneity. Data augmentation was used for effective training of the deep 3D network. BraTS 2018 dataset was used for experiments on the proposed architecture and compared with 2D CNNs. They reported an overall accuracy of 96.49% and concluded that data augmentation and suitable preprocessing could lead to better classification results. However, 3D models are computationally and memory-intensive methods thus, it would be better to have equivalent or better results using less computationally and memory-intensive methods. Kumar (2020) proposed an optimized deep learning algorithm Dolphin-SCA based Deep CNN to classify Glioma brain tumors from MR images [12]. Fuzzy deformable fusion with Dolphin Echolocation based Sine Cosine Algorithm (Dolphin-SCA) is used for segmentation. Features are extracted using power local directional patterns (LDP) and statistical features. Deep CNN is then used for the classification. The BraTS Q7 and SimBraTS datasets are used. The maximum accuracy achieved is 96.3%. However, this method does not take more features which might prove to be useful and does not classify the tumors to malignant and benign. In addition, the use of feature extraction along with deep CNN seems to add more unneeded complexity as deep CNN is capable of extracting features on its own. In [13], a multi-model CNN based hybrid approach is proposed for the classification of brain MR images. Similarly, several recent studies are discussed in [14] which utilizes different CNN models for brain tumor classification.

Overfitting often occurs due to excessively complex models containing large numbers of hidden layers. The model starts to learn noises in the training set that negatively affect the training data. Overfitting can also occur due to focus on the training set and building complex relations between features which might not work well with the new test data. The famous CNN models (LeNet, AlexNet and GoogleNet) lead to overfitting and do not perform well for brain tumor classification because of complex architectures with a high number of layers designed for many output classes (1000 classes) with RGB input images. AlexNet consists of a total of 25 layers and has more than 61 million parameters [15,16]. Similarly, GoogleNet consists of 144 layers and has more than seven million parameters [17,18]. In [19], an efficient brain tumor classification frame work is proposed where brain MR images are preprocessed to avoid overfitting problem.

## 2. Material and Methods

**Figure 5:**
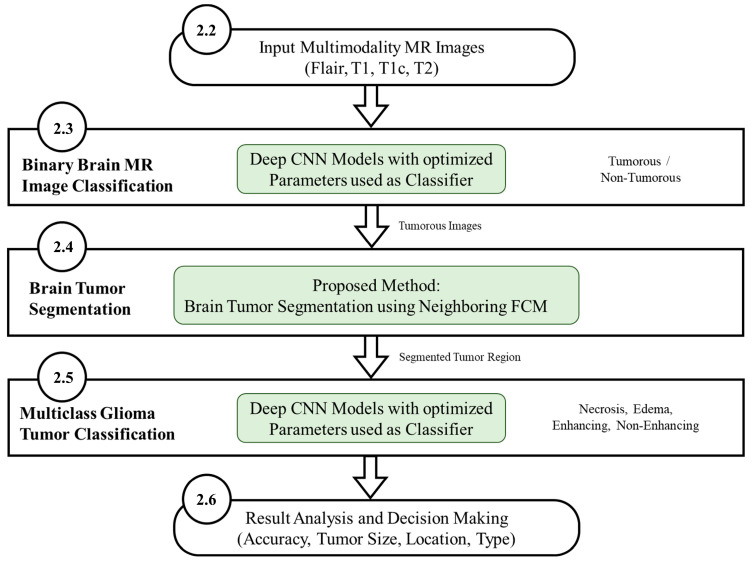
Workflow diagram of the proposed methodology (Green highlighted sections represent contributions in this work).

The workflow diagram of the new proposed methodology is shown in Figure 5. The multistage process consists of binary MR image classification for classifying the MR images into tumorous and non-tumorous using proposed CNN models, tumor segmentation to extract the tumorous region from the tumorous images, and multiclass Glioma tumor classification for classifying the Glioma tumors into four types. The use of deep learning-based CNN architecture classifies MR images producing an accuracy level superior to other techniques such as LeNet, AlexNet and GoogleNet.

Deep learning methods are gaining popularity in many areas of computer vision specifically image processing and speech analysis [20]. Deep learning can be used for classification, segmentation, and recognition in supervised learning [21]. Deep learning networks work by applying different sequential operations on the input data that transform the input such as convolution, sigmoid function application, etc. Each operation is performed in one layer of the deep network. The rectified linear activations (ReLU), residual connections, and an increased number of hidden layers make the performance better than classical neural networks. Another advantage of using deep learning networks is the availability of large datasets for training and the inherent data parallelism in the training process of the system which allows the use of modern-day GPUs for optimizing the system performance having millions of parameters. There are three major factors involved in using CNN for solving image processing problems. The architecture of the network, the regularization techniques used, and the optimization algorithms which are used in the training of the CNN system.

### 2.1. Experimental Environment

A dedicated 64-bit Windows 10 operating system machine equipped with GTX 1080 GPU having 2560 CUDA cores and 8 GB GDDR5X GPU memory was used for the experiments. The machine also contains 32-gigabyte memory (RAM) and a 3.70 GHz core i7 CPU. The software programs for pre-processing and segmentation were developed using MATLAB 2022a. Training and testing of MR images for classification into tumorous and non-tumorous images as well tumor classification among four different tumor types using CNN was performed using Python-based libraries called Keras, Tensor Flow, and Anaconda.

### 2.2. Experimental Datasets

For MR image classification, datasets from different sources were used. The gathered data sets are indicated in Table 1. For experiments, BraTS 2015 [22] dataset was mainly used along with the other datasets collected from different sources as described in Table 1. The dataset is split into training and testing with 80% and 20%, respectively for glioma tumor classification. Additionally, 20% of the training portion was used for cross-validation. The BraTS 2015 was used as a newer version of the BraTS because the BraTS 2018 dataset is actually the same as the BraTS 2015 dataset in terms of images (training and validation) in which expert radiologists manually revise all the ground truth labels. The BraTS 2018 dataset contains 384 training and testing patients’ data of both Low-Grade Glioma (LGG) and High-Grade Glioma (HGG). As per the WHO reports, LGG is considered as grade 1 and grade 2 tumor while HGG is considered as grade 3 and grade 4 tumor [23].

- Grade I: The brain tissue is benign and cell appearance is like normal brain cells, which grow slowly.
- Grade II: The brain tissue is malignant and cell appearance is less like the normal brain cells.
- Grade III: The brain tissue is malignant and appearance is very different from normal cells which are actively growing.
- Grade IV: The brain tissue is malignant and has the most abnormal appearance as compared to normal cells which grow rapidly.

**Figure 6:**
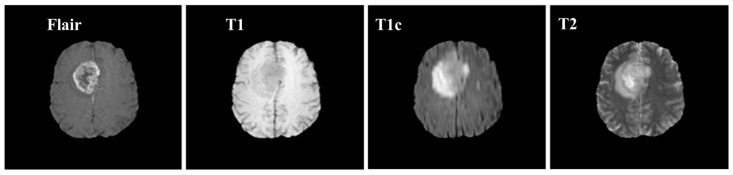
Sample MR images of T1, T2, T1c and Flair modalities.

Samples of these four modalities (T1, T2, T1c, Flair) are presented in Figure 6. All four modalities have 620 MR images which make a total of 239,320 MR images for all 384 cases and a total of 169,880 MR images 274 train images as shown in Table 2. In BraTS dataset, labels are provided only for the train images so only train images are used for experiments. The dataset is divided into 60% for training, 20% for validation and 20% for the testing. BraTS provides data in MetaImage (.mha) format which is used to store 3D medical images. For each modality of every case, there are 155 slices with 240 × 240 pixel dimensions which are stored in a single mha file.

**Figure 7:**
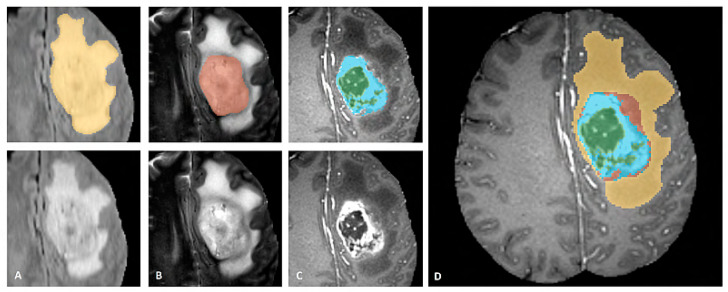
Sample of Multiclass Glioma labels with different modalities. The image label shows: (**A**). Whole tumor, (**B**). Tumor core, (**C**). Enhancing Tumor and (**D**). Combined all tumor types [22].

BraTS dataset provides annotations for the training cases with four different classes (Necrosis, Edema, Enhancing, and Non-Enhancing) and the fifth class is considered as everything else. The dataset is also described in three sub-compartment regions. Region 1 is known as Complete Tumor with labels 1, 2, 3, 4 in the annotated data. Region 2 is known as Tumor Core with labels 1, 3, 4, and Region 3 which is known as Enhancing Tumor with label 4 in the annotated data. The description of data labeling is presented in Table 3 and Figure 7 shows a sample of the labeled multiclass tumor in MR image. In Figure 7, the yellow color represents the whole tumor, red represents the core tumor, light blue represents enhancing tumor and green patches show the necrotic core [22].

In the preprocessing phase of the dataset, the 3D DICOM images were converted to PNG 2D images, and the metadata about patient information was removed. Each patient case includes five DICOM images (four DICOM for four MRI modalities; T1, T1c, T2 and Flair while the fifth DICOM image contains the annotation of the Glioma Tumor classes). Each DICOM contains 155 slices of grayscale MR images for a single patient. All images of the dataset are labeled based on the ground truth values provided by the annotations in the original BraTS dataset.

### 2.3. Binary Brain MR Image Classification

In this first stage, the binary classification is performed by inputting the images directly to the proposed deep CNN models. This is referred to as binary classification throughout the research. The experimental results are compared with the latest feature-based techniques such as texture features, block features and deep CNN as classifier-based techniques for example LeNet, AlexNet and GoogleNet.

#### 2.3.1. Typical Deep CNN Architecture

**Figure 8:**
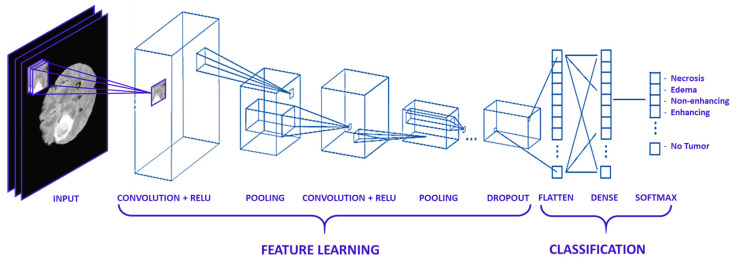
Typical CNN Architecture MR Image Classification.

In this work, a collection of parallel feature maps was formulated using different kernels that were slid over the input dataset. These are stacked together in the convolutional layer. While creating feature maps, a smaller dimension was used that helps in feature sharing between different layers. Kernel overlapping was avoided using zero-padding of the input images, which helps in managing the dimension of the convolution layer as well. A weighted sum of the input was passed through an activation function that helps determine which neuron should be rejected. The neurons having a higher weight associated with them are most probably to be rejected. Various activation functions are proposed in the literature for different types of deep learning applications, e.g., Linear, Sigmoid, ReLU, and softmax, etc. The pooling layer was applied after the convolution and non-linear transformation of the input dataset. In pooling Layers, the data is down-sampled to remove noise, smooth the data, and prevent overfitting. The data points which were extracted from the pooling layers were extended into column vectors. These column vectors were then used as input to a classical deep neural network. The architecture of the typical CNN Model for MR image classification is given in Figure 8.

#### 2.3.2. CNN Optimization Parameters for MR Images Classification

The proposed binary CNN architecture model 1 is described in Table 4 which consists of 9 layers and a total of 217,954 trainable parameters. An improved binary CNN architecture model 1 particularly for brain MR image classification is described in Table 5 which consists of a total of 10 layers but slightly fewer number of trainable parameters. After an intensive literature review of the optimization techniques of the CNN models and thorough study of the existing CNN models, enhanced CNN models are proposed. Several experiments were also performed using the existing models such as LeNet, AlexNet and GoogleNet. Similarly, different custom-built CNN models based on number of layers and parameters were tested to select the best combination of layers and parameters that perform better than existing CNN Models for the special nature of the grayscale MR images.

In model 2, the number of layers were increased to 10 layers as compared to 9 layers in model 1 but the number of parameters were reduced to 80,243 from 217,954 as shown in Table 4 and Table 5. In MR images, the tumor regions appear brighter compared to normal brain cells. Commonly, in an MR image, there is one tumor that appears in a particular shape inside the brain, which means that the more useful features can be found locally by keeping the convolutional filter size small. Another benefit of using small filter size for convolutional layers is weight sharing for all the pixels within the convolutional filter to extract local features from the brain MR images. Although Model 1 perform better than LeNet, AlexNet and GoogleNet but Model 2 further improves the performance by reducing the convolutional filter size and number of parameters.

### 2.4. Brain Tumor Segmentation

In the second stage, tumor regions were extracted from the tumorous images. Segmentation of brain tumor is a very complex task because of the complex anatomy of the brain structure [24]. Due to the low contrast and correlated MR scans, the segmentation task becomes highly complicated. For a comprehensive analysis of brain tumors from MR images, different patterns of effective parts of the brain are required through which the tumorous part can be differentiated from the rest of the brain. A brain can be divided into three main parts; Cerebrospinal Fluid (CSF), Gray Matter (GM), and White Matter (WM) [25]. The important task during the segmentation of brain MR images is to partition these tissues correctly. Hence, voxels’ labeling for specific tissue types carries immense importance in MR image segmentation [26]. As described earlier, the low contrast of brain MR images is another issue due to which it is difficult to differentiate among these three tissue types. The brain tissue overlapping issue is mainly addressed using FCM-based brain tumor segmentation technique.

In the proposed method tumor regions were extracted from the tumorous images by ignoring the non-tumorous images using the proposed neighboring FCM technique. To enhance the segmentation, the image intensity values were manipulated and tumor region was extracted using neighboring image features along with the actual image features. The tumor region was further enhanced by applying a region-growing algorithm. Brain tumor segmentation is useful for the identification and diagnosis of various types of tumors.

For proper diagnosis and proper treatment plan, the tumor must not only be detected but additional information such as tumor class, size and location should be identified. The tumorous portion of the image should be segmented in order to prepare the data for a second phase of classification to identify tumor class, size and location. It should be noted that segmentation is also a complicated step due to the complex nature of the image and the overlapping tissues and layers in the brain MR image.

**Figure 9:**
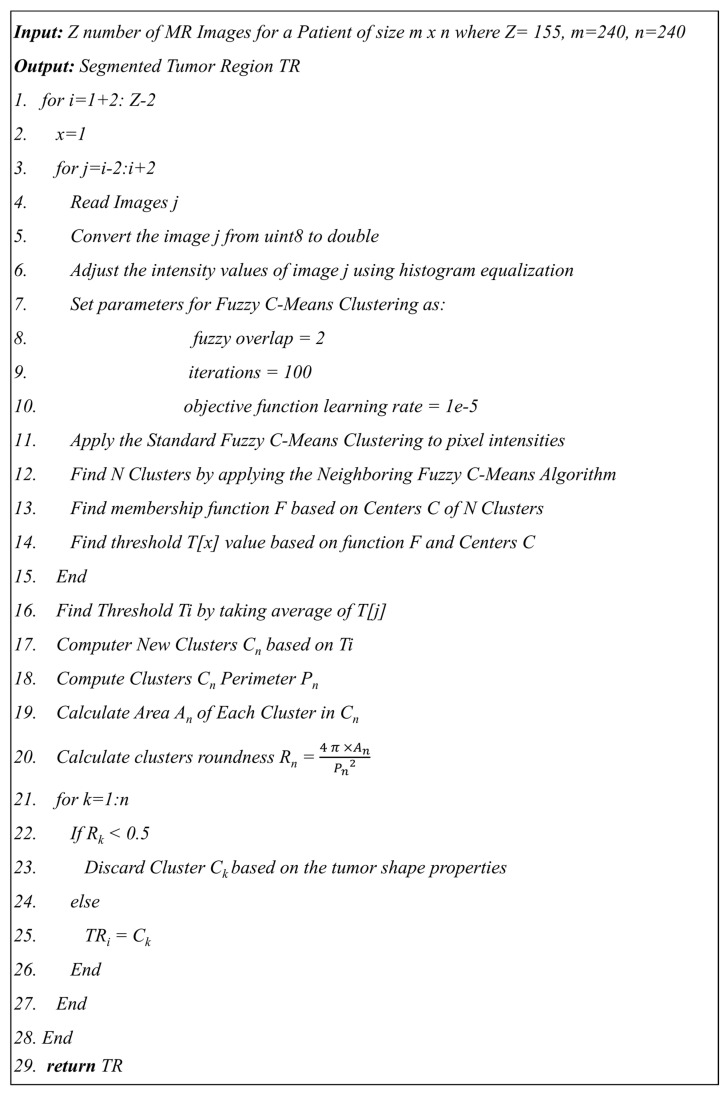
Proposed Algorithm 1 for tumor segmentation using Neighboring FCM.

The proposed segmentation method using neighboring FCM has an advantage over hard segmentation in that it retains more information from the original image. In this method, tumor regions were extracted using FCM from the tumorous images by ignoring the non-tumorous images. Figure 9 shows the proposed algorithm 1 used to find the tumor regions from the MR image using neighboring FCM.

In the proposed neighboring FCM, the standard FCM equation is modified to calculate the optimized centroid $y_{p}$ by including the previous two and next two images along with the actual image $x_{i}$ as shown in Equation (1).

(1) yp=∑p=1cuipq.(∑j=i−2i+2xj)5∑p=1cuipq

where $u_{ip}$ represents the membership value of a pixel located at the position i of class p. The $x_{j}$ is image intensity calculated based on the average of two previous images and two next images at position i instead of using single image intensity value at position i. The total number of classes is pre-defined c. The operator norm ‖.‖ represents the Euclidean distance and q represents the weightage for each fuzzy membership related to a specific class.

### 2.5. Multiclass Glioma Tumor Classification

In the final stage, a multiclass tumor classification is performed and an optimization driven deep CNN model is proposed as an enhanced brain MR image classification technique which categorizes the brain tumor into four types, i.e., Necrosis, Edema, Enhancing and Non-Enhancing. Before applying deep CNN. The experiments are compared with the latest CNN models such as LeNet, AlexNet and GoogleNet. The use of the deep CNN architecture classified images accurately producing higher accuracy than other techniques on the same database.

In this stage, multiclass tumor classification was performed and a deep CNN architecture was proposed as an enhanced brain MR image classification technique which categorizes the brain tumor into four types, i.e., Necrosis, Edema, Enhancing and Non-Enhancing. The use of the deep CNN architecture classified images accurately producing accuracy levels superior to other techniques on the same database. An enhanced CNN model 3 with 11 hidden layers and a total of 241,624 trainable parameters was proposed in Table 6 for multiclass Glioma tumor classification. The results were also compared with model 2 proposed for MR image classification presented in Section 3.3 and detailed in Table 5. In MR images, there is a strong association between the tumor tissues of different Glioma types (Necrosis, Edema, Enhancing, and Non-Enhancing) compared to the association between the tumorous tissues and non-tumorous (healthy) tissues of the brain. In CNN architecture, the lower convolutional layers mainly obtain intensity and shape features from the tumorous MR images while deeper features were extracted from feature maps which are more abstract and useful for the correct classification of the multiple types of Glioma tumor. Due to this fact, model 3 was proposed where the number of layers was increased along with the number of parameters to extract more useful features from the highly associated pixel values of tumorous region in the MR images.

### 2.6. Results Evaluation Techniques

The proposed framework is based on different steps and in order to quantify, the performance metrics; accuracy, precision, recall, F measure and Dice Similarity Coefficient (DSC) based on experiments are conducted for each step. The performance of the proposed method is measured based on True Positive (TP), False Positive (FP), True Negative (TN), and False Negative (FN) [24]. The percentage of predicted positive true cases that are in fact true positive is referred as precision. The rate of correctly predicted true positive to all the actual class observation is referred to as Recall. Furthermore, both Precision and Recall are used in the computation of the F Measure. DSC is a measurement in the spatial domain of the percentage of overlapped segmented portions of any two images.

## 3. Experimental Results and Discussions

The final output of the proposed system should be able to specify whether an image contains a tumor or not. For images that contain a tumor, the system further segments the tumorous region and classifies the tumor into one of four classes; Necrosis, Edema, Enhancing and non-enhancing. The system further specifies based on analysis the location and size of the tumor. The information retrieved from the system outperforms previous methods mentioned in the literature in terms of accuracy, precision, recall and F measure which helps to determine a proper diagnosis and proper treatment.

### 3.1. Brain MR Image Classification Results

Several experiments with different parameter combinations of batch size (100, 200, and 500) and epochs (4, 8, 16, 32, and 64) were performed for the proposed CNN models. The batch size of 100 and epochs value of 8 was found to be achieving the best accuracy and thus chosen with results presented. As shown in Table 7 and Table 8, the accuracy, precision, recall, and F-measure for model 1 and model 2 are presented. The results are also compared with the existing well-known CNN models like LeNet [27], AlexNet [15,16], and GoogleNet [17,28]. The AlexNet experiments show promising results with an accuracy of 96.95% and 96.53% for HGG and LGG, respectively as compared to LeNet and GoogleNet, but model 2 performance is far better than AlexNet, LeNet and GoogleNet. The famous CNN models (LeNet, AlexNet, GoogleNet) leads to overfitting and do not perform well for brain tumor classification because of complex architectures with high number of layers designed for very large number of output classes (1000 classes) with RGB input images. For example, AlexNet has 64 filters in the first convolutional layer which are mostly encoded with color information. Due to the small batch size for a large dataset of 169,880 MR images with enhanced CNN model 2, highest results are achieved with 8 epochs. It is also clear in the results that the number of Epochs is directly proportional to effectiveness. The effectiveness increases with the increase in the number of Epochs. The proposed models especially model 2 achieved the best accuracy for both LGG and HGG and for all modalities. For HGG, model 2 achieved the best classification accuracy for the flair modality of 98.74% with precision of 0.983, recall of 0.985, and F-measure of 0.984. For LGG, model 2 achieved the best accuracy for the flair modality of 97.33% with precision of 0.960, recall of 0.988, and F-measure of 0.974. Model 2 outperformed model 1 and outperform the well-known CNN models specified.

The proposed models are also tested on the AANLIB and PMIS datasets and results are compared with LeNet, AlexNet and GoogleNet CNN models as shown in Table 9. The results are reaffirmed and validated showing that for both AANLIB and PMIS datasets, the proposed CNN models outperformed the well-known CNN models and that Model 2 in outperformed all other models by achieving 100% accuracy. This shows that the proposed models solve the problems of overfitting as well as problems of data availability.

Table 10 shows a summary of the results obtained in this work and a comparison with the latest literature. The proposed methods achieved an average accuracy ranging from 96.88% to a maximum of 98.74%, whereas, previously published results indicated relatively less accuracies as shown in Table 10. The proposed CNN model 2 as classifier achieved 98.74% on a very large dataset (BraTS 2015). The accuracy obtained using the proposed approach in this work is very high even when using a big dataset (BraTS 2015), which shows the robustness of the approach.

### 3.2. Glioma Tumor Segmentation Results

Tumor regions were extracted from the tumorous images by ignoring the non-tumorous images using the proposed neighboring FCM based tumor segmentation method. The experimental results of brain tumor segmentation are evaluated based on visual comparison, accuracy, specificity, sensitivity, dice similarity coefficient (DSC) and mutual information (MI).

**Figure 10:**
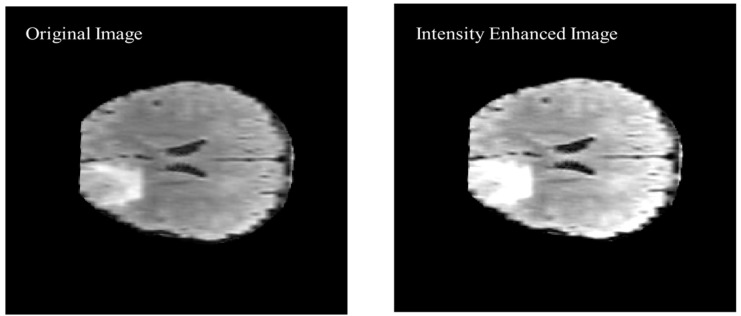
Visual comparison of the original image and intensity enhanced image.

As shown in Figure 9 of the proposed algorithm, image intensity values were manipulated to enhance the segmentation by saturating the highest 1% and lowest 1% of all the pixel values in the MR image which enhances the contrast of the grayscale image. The enhancement method was applied to all the images before converting the image to black and white. The visual results are shown in Figure 10 to compare the original brain MR sample image and the intensity manipulated image.

**Figure 11:**
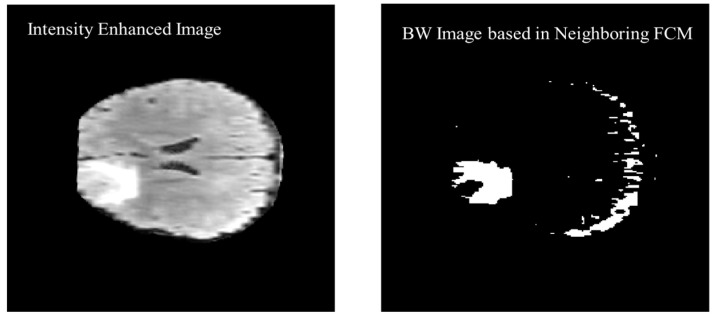
Black and White (BW) binary image generated based on the neighboring FCM threshold applied to intensity enhanced image.

The previous two and following two images are used as a reference along with the actual image to calculate the threshold value for the segmentation. Figure 11 shows the black and white (BW) binary image generated based on the neighboring FCM threshold applied to intensity-enhanced MR image.

**Figure 12:**
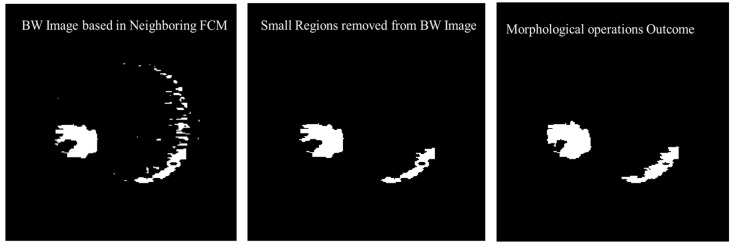
Visual results after removing the small regions from the binary image and applying the morphological operations.

Morphological operations are applied for further enhancement of the tumor region in the binary image. Small regions in the binary are removed based on the connected pixel count values of less than 256 from the 240×240 MR image having total 57,600 pixels. Erosion and dilation morphological operations with a structure size of 2×2 pixels are applied to fill the small gaps in the binary image. Figure 12 shows the visual results after removing the small regions from the binary image and applying the morphological operations (erosion and dilation).

**Figure 13:**
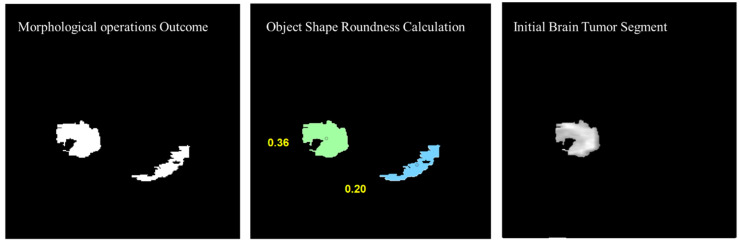
Object shape roundness calculation and generating the initial tumor segment.

To remove the non-tumor parts from the binary image, the number of objects was calculated in the binary image and the tumor region was selected based on the shape roundness properties of the objects. In Figure 13, objects’ roundness properties are measured from the binary image and the roundness values for each object are displayed. The initial brain tumor segment is extracted based on the best roundness value.

**Figure 14:**
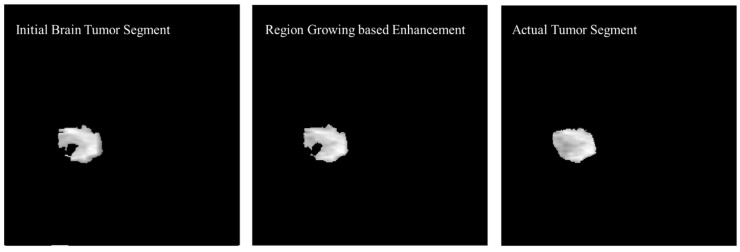
Enhancement in the initial brain tumor segment by applying region growing method and visual comparison with the actual tumor segment.

The initial tumor segment is enhanced using the region growing method. The first step in the process of edge segmentation based on the region-growing technique is to find the seed pixels which is selected based on the Neighboring FCM based initial segmentation. In the first step, a geometric structure from a gray level image was secured then centers of adjacent labeled edges were given as initial input to the algorithm. Figure 14 shows the enhancement in the initial brain tumor segment by applying region growing method and a visual comparison was made with the actual tumor segment.

**Figure 15:**
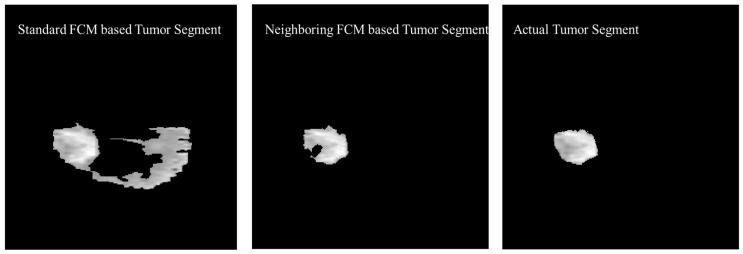
Visual Comparison of the Standard FCM based tumor segment with the Neighboring FCM based tumor segment and the actual tumor segment.

The experimental results were also generated based on the standard FCM to compare with the proposed neighboring FCM based tumor segmentation method. Figure 15 shows the visual comparison of the standard FCM based tumor segment with the neighboring FCM based tumor segment and the actual tumor segment.

Better effectiveness of neighboring FCM can be observed through statistical analysis. Prominent improvement of 14.3% and 16.37% was secured with respect to Accuracy and DSC, respectively and other parameters as well. In the proposed neighboring FCM technique, the labeling of the images is going to be influenced by immediate neighbors only, so it gives better results as compared to standard FCM. The proposed method has outperformed in terms of average DSC, specificity, and sensitivity with values 90.87%, 99.86% and 95.52% as compared to the standard FCM average DSC, specificity, and sensitivity with values 66.86%, 87.22% and 81.29%, respectively.

Table 11 shows a comparison between the proposed segmentation technique with techniques proposed in recent literature. Dice Similarity Coefficient (DSC) is used for comparison purposes as it is the common metric adopted in recent literature. Results indicate that the proposed segmentation technique achieved an average DSC of 90.87% which outperforms all the methods listed in Table 11.

The reason for this improvement was mainly because of extra neighboring information incorporated along with the original image and in the final stage region growing algorithm was used to secure a more accurate segmented image. FCM parameter selection is highly sensitive to noise and computational time will increase rapidly with non-homogeneous pixel intensities. The modification in original FCM function is made to tackle the non-homogeneous intensities of the pixels. In the proposed method, each image is influenced by immediate neighbors. This phenomenon creates a regularized effect and influence on labeling with respect to neighbors, which will secure a more homogeneous biased solution.

### 3.3. Glioma Tumor Classification Results

Although Model 2 was proposed for binary classification of MR image into tumorous and nontumorous it still performed better than LeNet, AlexNet and GoogleNet for multiclass Glioma tumor classification as shown in Table 12 and Table 13 but the Model 3 further improved the performance by reducing the convolutional filter size and number of parameters. The CNN architecture models were proposed namely; model 2 which is same model proposed and explained in Section 3.3 (Table 6) and model 3 which is optimized model architecture described in Table 7. As shown in Table 12 and Table 13, model 2 achieved highest average accuracy 95.94% and 96.30% for HGG and LGG, respectively using Flair images. The combination of proposed enhanced model 3 and Batch size 200, epochs 8 reduced the overfitting and improved the classification accuracies as compared to other well-known CNN architectures; LeNet, AlexNet, and GoogleNet. It also outperformed model 2 proposed in this study.

In the proposed enhanced CNN model 3 was used as classifier and parameters such as batch size and epoch were further tuned based on accuracy results. The results of the proposed model 3 were compared with the existing CNN models (LeNet, AlexNet and GoogleNet) and model 2. For HGG Glioma, model 3 with batch size of 200 and 8 epoch achieved the highest average accuracy of 95.94% for enhanced model 3 with Flair MR images. The average accuracies showed improvement as shown in Table 12 and Table 13. For LGG Glioma classification, the batch size of 200 and 8 epoch secured the highest average accuracy for model 3 of 96.30% accuracy for Flair MR images.

The AlexNet experiments show promising results with an average accuracy of 92.31% and 93.14% for HGG and LGG, respectively as compared to LeNet and GoogleNet but still model 3 performance is far better than AlexNet, LeNet and GoogleNet. The famous CNN models (LeNet, AlexNet, and GoogleNet) do not perform well for brain tumor classification because of the complex architectures with high number of layers and parameters designed for very large number of output classes (1000 classes) with RGB input images. For example, AlexNet has 64 filters in the first convolutional layer which are mostly encoded with color information. In a deep learning model, if the number of parameters is higher than the training data set as observed for the case of LeNet, AlexNet and GoogleNet. In this case, regularization becomes a more critical step. The approximation of temporary functions of the input data in the design of CNN architecture plays an important role. This approximation is connected with the selection of parameters of the network like depth and width. In the process of regularization, overfitting of the algorithm is avoided especially when the complexity of the model increases. Hence, from the statistical analysis presented in Table 12 and Table 13, it can be concluded that if CNN model 3 is used as a classifier then the best classification accuracy is achieved for all the classes and Glioma types with a batch size of 200 and 8 epochs.

As shown in Table 14, the proposed technique using CNN as a classifier achieved an accuracy of 96.30% for multiclass classification. When compared with other recent techniques from literature, it is evident that the proposed technique outperformed those listed in Table 14. This also outperformed the other methods that were published recently using the same dataset.

## 4. Conclusions and Future Work

A brain tumor is deadly and painful disease. It can lead to death if not diagnosed in its early stages. Manual extraction of tumor segments by doctors is a time-consuming and irreversible process. In this study, a framework named DeepTumor is presented for the multistage-multiclass Glioma tumor classification into four classes, edema, necrosis, Enhancing and Non-enhancing. A multistage automated brain tumor classification method was proposed with high accuracy that can assist radiologists in accurate and early diagnosis of the brain tumor. The experiments were performed using multimodality (Flair, T1, T1c, T2) BraTS 2015 MRI dataset. The first stage, the proposed CNN classifier model 2 achieved a 98.74% accuracy for High-Grade Glioma (HGG) and 97.33% accuracy for Low-Grade Glioma (LGG) MR Image classification. In the second stage, the tumorous portion of the image was segmented using an enhanced proposed technique that uses the neighboring images Fuzzy C-means (FCM) information along with the actual image to perform the tumor segmentation. By using this technique, the tumor region information was extracted with a higher accuracy rate. In the third stage, segmented tumors were classified into four Glioma tumor classes; Necrosis, Edema, Non-enhancing tumor, and enhancing tumor. The experimental results showed that for multiclass tumor classification, an average accuracy of 96.30% was achieved using Deep CNN Classifier.

As future work, an automated decision support system can be integrated. The system will provide intelligent decisions for doctors by analyzing the size, shape, location, and type of the tumor by predicting the prevalence rate, the severity of brain cancer, and surgery decisions. The size and type of the Glioma brain tumor is a direct indicator of the tumor grade and the severity of brain cancer. As the structural and spatial parameters of brain tumors like size, shape, and location play an important role in radiologists’ decisions, future work can include methods to approximate the volume of the brain tumor and create a 3D model. In future work, machine learning algorithms with combined CNN features from MR images and radiomic features can be used for the prediction of patient survival. Additionally, the proposed method will continue to be enhanced to further achieve higher accuracies in brain tumor segmentation and classification as well as applying the same method and its enhancement on other medical conditions such as skin cancer.

## Figures and Tables

**Table 1:** Brain MRI Dataset Description.

Data Source	MRI Type	Slice Thickness (mm)	Number of Patients	Number of Images in Each Patient Data
MICCAI BraTS MRI Dataset [22]	T1, T1c T2, Flair	5.0	384	155 Scans
PIMS-MRI Dataset [5]	T1, T2	5.0	8	86 to 210 Scans
Harvard Medical School Dataset AANLIB [5]	Flair	5.0	1	90 Scans

**Table 2:** Summary of Acquired BraTS MRI Dataset.

BRATS (2015/2016) Dataset Description		
Total Number of Cases	384	
Total Number of MR Images	239,320	
Modalities for Each Case	4 (T1, T2, T1c, and T2 Flair)	
MR Image Pixel Resolution	240 × 240	
MR Image in each Modality	155	
Total MR Image for each Case	620	
Training Datasets	274	
	LGG (Training Cases)	54
	HGG (Training Cases)	220
	Total Annotation Images	42,470
	Total Training MR Images	169,880
Testing Datasets (Combined LGG & HGG)	110	
	Total Testing MR Images	68,200

**Table 3:** BraTS Annotations and Sub Compartments.

Tumor Class Labels		Sub Compartments		
Tumor Type	Data Label	Regions	Label ID	Type
Necrosis	1	Region 1	1 + 2 + 3 + 4	Complete Tumor
Edema	2	Region 2	1 + 3 + 4	Tumor Core
Non-enhancing Tumor	3	Region 3	4	Enhancing Tumor
Enhancing Tumor	4	No Tumor		
Everything Else	0			

**Table 4:** Proposed CNN Model 1 for MR Image Classification.

#	Layer Name	Input Description	Output Shape	Parameters
L1	Input	MR Images (30 × 30 × 1)	30 × 30 × 1	0
L2	Convolution 1	Filters (16.5 × 5), (30 × 30 × 1)	26 × 26,16	16 × 5 × 5 + 16 = 416
L3	Max Pooling 1	Pooling of 2 × 2	13 × 13,16	0
L4	Dropout 1	20% Dropout	13 × 13,16	0
L5	Max Pooling 2	Pooling of 2 × 2	7 × 7.16	0
L6	Flatten	Convert 7 × 7.16 to Linear	784	0
L7	Dense 1	ReLU based Dense Layer	256	784 × 256 + 256 = 200,960
L8	Dense 2	ReLU based Dense Layer	64	64 × 256 + 64 = 16,448
L9	Dense 3	ReLU based Dense Layer	2	2 × 64 + 2 = 130
Total Trainable Parameters: 217,954				

**Table 5:** Proposed Improved CNN Model 2 for MR Image Classification.

#	Layer Name	Input Description	Output Shape	Parameters
L1	Input	MR Images of size (30 × 30 × 1)	30 × 30 × 1	0
L2	Convolution 1	Filters (30.3 × 3), (30 × 30 × 1)	28 × 28.30	30 × 3 × 3 + 30 = 300
L3	Max Pooling 1	Pooling of 2 × 2	14 × 14.30	0
L4	Convolution 2	Filters (15.3 × 3), (14 × 14 × 30)	12 × 12.15	15 × 3 × 3 × 30 +15 = 4065
L5	Max Pooling 2	Pooling of 2 × 2	6 × 6.15	0
L6	Dropout 1	20% Dropout	6 × 6.15	0
L7	Flatten	Convert 6 × 6.15 to Linear	540	0
L8	Dense 1	ReLU based Dense Layer	128	128 × 540 + 128 = 69248
L9	Dense 2	ReLU based Dense Layer	50	50 × 128 + 50 = 6528
L10	Dense 3	ReLU based Dense Layer	2	2 × 50 + 2 = 102
Total Trainable Parameters: 80,243				

**Table 6:** Proposed CNN model 3 specifically for Glioma tumor classification.

#	Layer Name	Input Description	Output Shape	Parameters
L1	Input	MR Images of size (30 × 30 × 1)	30 × 30 × 1	0
L2	Convolution 1	Filters (30.3 × 3), (30 × 30 × 1)	28 × 28.30	30 × 3 × 3 + 30 = 300
L3	Max Pooling 1	Pooling of 2 × 2 on 28 × 28,30	14 × 14.30	0
L4	Convolution 2	Filters (60.3 × 3), (14 × 14 × 30)	12 × 12.60	60 × 3 × 3 × 30 +60 = 16,260
L5	Dropout 1	20% Dropout	12 × 12.60	0
L6	Convolution 3	Filters (30.3 × 3), (12 × 12 × 60)	10 × 10.30	30 × 3 × 3 × 60 +30 = 16,230
L7	Max Pooling 2	Pooling of 2 × 2 on 10 × 10.30	5 × 5.30	0
L8	Flatten	Convert 5 × 5.30 to Linear	750	0
L9	Dense 1	ReLU based Dense Layer	256	256 × 750 + 256 = 192,256
L10	Dense 2	ReLU based Dense Layer	64	64 × 256 + 64 = 16,448
L11	Dense 3	Softmax based Dense Layer	2	2 × 64 + 2 = 130
Total Trainable Parameters: 241,624				

**Table 7:** Classification results using proposed CNN Models as classifier and comparison with existing CNN models for brain HGG MR images.

CNN Modal	Modality	Accuracy	Precision	Recall	F Measure
Proposed Model 1	Flair	96.88	0.961	0.989	97.990
	T1	93.55	0.917	0.958	0.937
	T1c	95.38	0.942	0.967	0.954
	T2	96.15	0.984	0.938	0.961
Proposed Model 2	Flair	98.74	0.983	0.985	0.984
	T1	94.51	0.928	0.965	0.946
	T1c	95.47	0.961	0.948	0.954
	T2	96.34	0.969	0.958	0.963
LeNet	Flair	87.31	0.872	0.848	0.860
	T1	82.63	0.762	0.865	0.811
	T1c	85.61	0.798	0.890	0.842
	T2	87.18	0.846	0.857	0.852
AlexNet	Flair	96.95	0.972	0.961	0.967
	T1	92.64	0.873	0.969	0.919
	T1c	92.39	0.883	0.948	0.915
	T2	95.37	0.943	0.950	0.946
GoogleNet	Flair	94.13	0.922	0.952	0.937
	T1	87.51	0.845	0.869	0.857
	T1c	86.93	0.804	0.919	0.858
	T2	89.74	0.873	0.890	0.882

**Table 8:** Classification results using proposed CNN Models as classifier and comparison with existing CNN models for brain LGG MR images.

CNN Modal	Modality	Accuracy	Precision	Recall	F Measure
Proposed Model 1	Flair	96.29	0.962	0.964	0.963
	T1	96.88	0.954	0.985	0.969
	T1c	95.25	0.961	0.944	0.952
	T2	94.66	0.952	0.941	0.946
Proposed Model 2	Flair	97.33	0.960	0.988	0.974
	T1	95.55	0.942	0.970	0.956
	T1c	95.55	0.930	0.985	0.957
	T2	96.29	0.970	0.955	0.963
GoogleNet	Flair	83.75	0.822	0.780	0.801
	T1	86.85	0.843	0.843	0.843
	T1c	82.38	0.808	0.760	0.783
	T2	86.97	0.819	0.884	0.850
AlexNet	Flair	96.53	0.964	0.953	0.958
	T1	95.04	0.921	0.964	0.942
	T1c	93.42	0.908	0.938	0.923
	T2	94.91	0.963	0.914	0.938
GoogleNet	Flair	90.20	0.889	0.875	0.882
	T1	89.58	0.852	0.908	0.879
	T1c	83.75	0.755	0.905	0.823
	T2	90.45	0.892	0.878	0.885

**Table 9:** Experimental Results for Validation Datasets (PMIS-MRI and AANLIB).

Dataset	Classifier	Accuracy	Precision	Recall	F Measure
AANLIB	Proposed Model 1	100	1	1	1
	Proposed Model 2	100	1	1	1
	LeNet	94.44	1	0833	0.909
	AlexNet	100	1	1	1
	GoogleNet	100	1	1	1
PMIS	Proposed Model 1	96.87	0.963	1	0.942
	Proposed Model 2	100	1	1	1
	LeNet	90.38	0. 950	0.826	0.884
	AlexNet	100	1	1	1
	GoogleNet	98.07	0.958	1	0.979

**Table 10:** Comparison of the proposed method for brain MR Images Classification with latest literature techniques.

Method	Data	Accuracy
Proposed Method (CNN as a Classifier–Model 2)	BraTS	98.74%
Proposed Method (CNN as a Classifier–Model 1)	BraTS	96.88%
Five CNN layers based Model [10]	BraTS	97.5%
Multi-Scale 3D CNN [11]	BraTS	96.49%
Optimization driven Deep CNN [12]	BraTS	96.3%
Hybrid CNN features and KNN [29]	BraTS	96.25%
Block Based Features and Random Forest Classifier [30]	BraTS	95%
GLCM Features, SVM [31]	251	85%

**Table 11:** Comparison of the proposed brain Tumor Segmentation method with latest literature techniques.

Method	Data	DSC
Proposed Method (Neighboring FCM + Region Growing)	BraTS	90.87%
Potential field based tumor segmentation [32]	BraTS	88% (±4%)
Preprocessing (Mean filter + Histogram equalization + Laplacian edge enhancement) and segmentation through Otsu Thresholding [30]	BraTS	84%
Gabor filter + Histogram equalization + Laplacian edge enhancement and segmentation through Otsu Thresholding [33]	BraTS	83%
No preprocessing and segmentation through Otsu Thresholding [33]	BraTS	70%

**Table 12:** Multiclass Glioma Tumor Classification results using proposed CNN models classifiers compared with other well-known CNN models for brain HGG MR images.

CNN Modal	Modality	Individual Accuracies				Average Measures			
		Necrosis	Edema	Non-Enhancing	Enhancing	Acc.	Precision	Recall	F Measure
Proposed Model 2	Flair	89.92	97.65	94.86	95.86	94.57	0.949	0.932	0.940
	T1	84.48	96.64	86.39	87.47	88.74	0.890	0.875	0.882
	T1c	87.10	98.99	83.82	87.27	89.30	0.889	0.884	0.886
	T2	88.25	96.64	85.13	86.92	89.24	0.910	0.861	0.884
Proposed Model 3	Flair	94.74	99.24	95.52	94.27	95.94	0.948	0.942	0.943
	T1	89.30	97.86	89.98	89.75	91.72	0.917	0.908	0.909
	T1c	88.39	97.65	86.00	89.40	90.36	0.922	0.870	0.894
	T2	90.73	97.99	86.19	89.67	91.15	0.936	0.873	0.901
LeNet	Flair	85.66	97.99	73.55	74.35	82.89	0.811	0.766	0.787
	T1	70.43	97.99	67.87	69.94	76.56	0.734	0.817	0.770
	T1c	75.50	98.32	67.39	71.91	78.28	0.772	0.773	0.768
	T2	76.07	97.99	73.41	77.87	81.33	0.789	0.825	0.805
AlexNet	Flair	90.83	99.66	87.15	91.60	92.31	0.920	0.895	0.907
	T1	87.39	98.32	84.14	88.88	89.68	0.886	0.868	0.877
	T1c	86.62	97.65	86.77	86.26	89.32	0.889	0.851	0.867
	T2	88.82	98.32	87.78	88.62	90.88	0.893	0.900	0.896
GoogleNet	Flair	74.73	97.99	75.93	77.64	81.57	0.791	0.828	0.809
	T1	76.02	96.64	76.13	76.09	81.22	0.801	0.801	0.801
	T1c	86.77	97.32	80.88	86.03	87.75	0.884	0.839	0.860
	T2	80.51	99.33	74.82	79.18	83.46	0.819	0.826	0.822

**Table 13:** Multiclass Glioma Tumor Classification results using proposed CNN models classifiers compared with other well-known CNN models for brain LGG MR images.

CNN Modal	Modality	Individual Accuracies				Average Measures			
		Necrosis	Edema	Non-Enhancing	Enhancing	Acc.	Precision	Recall	F Measure
Proposed Model 2	Flair	92.80	100.00	91.52	92.76	94.27	0.948	0.937	0.942
	T1	88.91	100.00	86.84	85.98	90.43	0.905	0.906	0.904
	T1c	89.69	98.25	86.26	91.36	91.39	0.915	0.917	0.916
	T2	92.61	96.49	93.86	91.36	93.58	0.931	0.939	0.942
Proposed Model 3	Flair	94.89	100.00	95.44	94.86	96.30	0.957	0.970	0.955
	T1	93.00	100.00	86.84	92.06	92.97	0.914	0.949	0.931
	T1c	93.39	100.00	92.11	92.06	94.39	0.942	0.947	0.944
	T2	92.80	98.25	87.13	92.52	92.68	0.958	0.899	0.924
LeNet	Flair	75.63	98.25	82.40	74.33	82.65	0.765	0.714	0.726
	T1	70.29	98.25	70.71	66.49	76.43	0.776	0.707	0.733
	T1c	66.79	98.25	64.57	61.11	72.68	0.702	0.767	0.729
	T2	76.32	98.25	64.57	74.43	78.39	0.793	0.717	0.751
AlexNet	Flair	90.90	100.00	92.49	89.18	93.14	0.954	0.831	0.879
	T1	87.02	98.25	85.04	82.37	88.17	0.858	0.890	0.872
	T1c	90.33	98.25	84.46	84.94	89.49	0.885	0.877	0.881
	T2	88.58	98.25	86.80	84.94	89.64	0.905	0.857	0.879
GoogleNet	Flair	81.54	98.25	82.55	79.67	85.50	0.867	0.722	0.757
	T1	80.60	100.00	78.02	72.33	82.74	0.813	0.810	0.811
	T1c	79.24	100.00	71.88	71.86	80.75	0.765	0.868	0.811
	T2	76.12	98.25	75.39	71.63	80.35	0.763	0.859	0.807

**Table 14:** Comparison of the proposed method for multiclass Glioma Tumor Classification with literature techniques.

Method	Data	Accuracy
Proposed Method (CNN as a Classifier–Model 3)	BraTS	96.30%
Texture Features from Supervoxels and Random Forest [34]	BraTS	90.67%
Ten Statistical Features and Random Forest [35]	BraTS	80.85%
Dual path Residual Convolutional Neural Network [36]	BraTS	84.90%
Deep CNN with extensive data augmentation [37]	BraTS	94.58%
Using morphological processing and classification using unified algorithm [38]	BraTS	95.97%
Multi-level attention network [39]	BraTS	94.91%

## Data Availability

The data used in this research was acquired from publicly.
